# SDWPF: A Dataset for Spatial Dynamic Wind Power Forecasting over a Large Turbine Array

**DOI:** 10.1038/s41597-024-03427-5

**Published:** 2024-06-19

**Authors:** Jingbo Zhou, Xinjiang Lu, Yixiong Xiao, Jian Tang, Jiantao Su, Yu Li, Ji Liu, Junfu Lyu, Yanjun Ma, Dejing Dou

**Affiliations:** 1 Business Intelligence Lab, Baidu Research, Beijing, China; 2 China Longyuan Power Group Corp. Ltd., Beijing, China; 3 Tsinghua University, Beijing, China; 4 Baidu Inc., Beijing, China; 5 BEDI Cloud, Beijing, China; 6 Fudan University, Shanghai, China

**Keywords:** Wind energy, Computer science

## Abstract

Wind power is a clean and renewable energy, yet it poses integration challenges to the grid due to its variable nature. Thus, Wind Power Forecasting (WPF) is crucial for its successful integration. However, existing WPF datasets often cover only a limited number of turbines and lack detailed information. To bridge this gap and advance WPF research, we introduce the Spatial Dynamic Wind Power Forecasting dataset (SDWPF). The SDWPF dataset not only provides information on power generation and wind speed but also details the spatial distribution of the wind turbines and dynamic contextual factors specific to each turbine. These factors include weather information and the internal status of each wind turbine, thereby enriching the dataset and improving its applicability for predictive analysis. Further leveraging the potential of SDWPF, we initiated the ACM KDD Cup 2022, a competition distinguished as the foremost annual event in data mining, renowned for presenting cutting-edge challenges and attracting top talent from academia and industry. Our event successfully draws registrations from over 2400 teams around the globe.

## Background & Summary

The estimation of wind power supply in advance, known as Wind Power Forecasting (WPF), can benefit diverse downstream applications, including power systems operations, maintenance scheduling, and profit maximization for power traders. Wind power plays a leading role in electricity production in the renewable energy sector due to its high efficiency, affordability, and environmental friendliness^1–3^. However, fluctuations and uncertainties in wind speed and direction pose significant obstacles to the increase of wind power penetration in the power grid. These fluctuations necessitate power substitution from other sources that might not be immediately available (e.g., it generally takes at least six hours to fire up a coal plant) to maintain the balance between electricity generation and consumption. Therefore, WPF has been widely recognized as one of the most critical issues in wind power integration and operation^4–7^. Nevertheless, carrying out WPF with high prediction accuracy is always demanded to ensure grid stability and supply security. Over the last decade, wind power has become one of the fastest-growing renewable energy sources globally^8^. Additionally, numerous studies have investigated wind power forecasting problems in recent years^9–15^.

Most datasets that underlie WPF research are not publicly accessible due to confidentiality agreements, as noted by^16^. Publicly available wind power datasets are typically aggregated over spatial regions, lacking turbine-level measurements and turbine-specific power output^17^. The Supervisory Control And Data Acquisition (SCADA) system is responsible for collecting turbine-level measurements, which reflect dynamic contextual factors for each turbine, such as temperature, weather, and turbine internal status. Previous studies^16,18^ have demonstrated that these turbine-level dynamic context factors, along with turbine locations, can help increase the accuracy of WPF models. Although SCADA data can be easily utilized by deployed WPF systems in the real world, non-confidential datasets with such information remain scarce. For instance, the popular Kaggle datasets^19,20^ only provide the information of one turbine, whose location and data origins are unknown. To the best of our knowledge, the dataset with the largest number of turbines is shown in^21^, which consists of only 32 turbines. However, a typical wind farm may have hundreds of turbines. A discussion about the related work of wind power forecasting data can be found in Supplementary Section A.

The absence of a large-scale, real-world public benchmark dataset may impede progress in WPF research. A real-world benchmark dataset plays a vital role in assessing the limits of existing methodologies, fostering technological advancements, and enhancing educational efforts in this domain. The significance of such a dataset can be detailed through three main aspects: (1) *Model Verification*: Public benchmark datasets establish evaluation standards for forecasting models, offering definitive guidelines for model selection and deployment. This standardization is essential for ensuring the accuracy and comparability of WPF methods. (2) *Research Advancement*: Benchmark datasets act as a proving ground for identifying and evaluating promising new technologies and models dedicated to WPF. By encouraging a competitive environment, these datasets drive research innovation and spotlight areas ripe for breakthroughs. (3) *Educational Value*: Benchmark datasets are also educational resources that equip new researchers and students with a deeper understanding of wind power forecasting and the complexities of model development. Thus, the availability of a WPF benchmark dataset is instrumental in advancing wind power forecasting, contributing to a sustainable energy future.

In this paper, we introduce a novel dataset for Spatial Dynamic Wind Power Forecasting, denoted as SDWPF. This dataset includes the spatial distribution of wind turbines, along with dynamic contextual factors derived from the SCADA system. SDWPF is constructed based on the data of a real-world wind power farm belonging to China Longyuan Power Group Corp. Ltd., the largest wind power producer in China and Asia. Compared to the previously available datasets, SDWPF has two distinct features: (1) Spatial distribution: it includes the relative location and elevation of all wind turbines in a wind farm to model the spatial correlation among them. (2) Dynamic context: The dataset provides weather information and the internal status of each wind turbine, as detected by the SCADA system, to facilitate the forecasting task.

In the SDWPF dataset, the wind farm’s turbine array consists of 134 wind turbines, representing a notable enlargement in array size compared to the existing largest dataset, which contains merely 32 wind turbines. The SDWPF dataset encompasses wind power production records sourced from the wind farm’s SCADA system. The dataset covers 24 months and contains more than 11.4 million historical entries detailing wind, temperature, and the internal status of each turbine. These records are collected at a 10-minute resolution from each turbine within the turbine array of the wind farm. We also detail the data collection process, explore the characteristics in-depth, and discuss potential caveats associated with using this data. Additionally, we conducted an ablation study to demonstrate the effectiveness of several data features.

To explore the performance limits of existing WPF methods and to promote research in wind power technology using machine learning techniques, we utilize the SDWPF dataset to launch the ACM KDD Cup 2022 Challenge, which has been the most prestigious annual data mining competition held in conjunction with the ACM SIGKDD conference. The information about the challenge is briefly introduced in Supplementary Section C.4 and is also available on the official website of the Baidu KDD Cup 2022^22^. The Baidu KDD Cup attracted over 2400 registration teams from around the world, some of which submitted cutting-edge models that produced significant improvements over our official baseline. It is our hope that the SDWPF dataset will foster the development of wind power forecasting research, contributing to a sustainable energy future.

## Methods

**Fig. 1:**
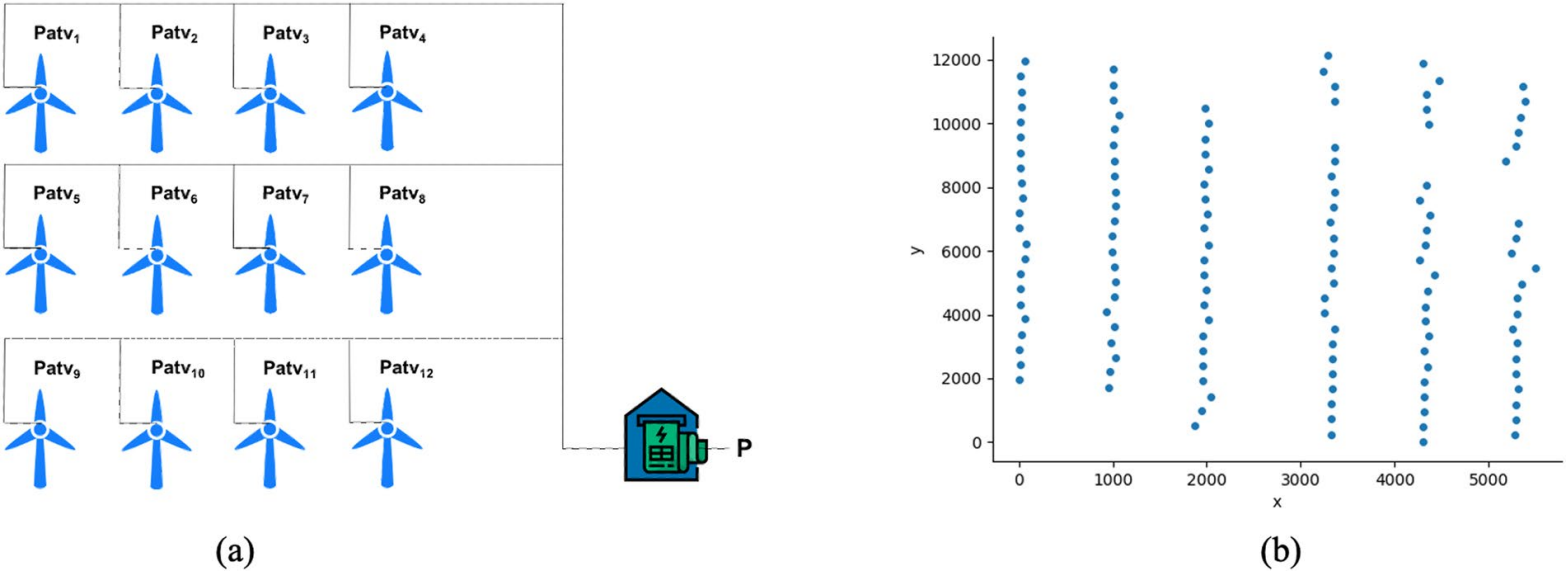
Illustration of a turbine array and its spatial distribution. (**a**) An illustration of a wind farm’s turbine array and its output power. (**b**) Spatial distribution of wind turbines (x and y are with the meter unit) in the SDWPF dataset.

The SDWPF dataset is collected from the Supervisory Control and Data Acquisition (SCADA) system of a wind farm. Each wind turbine can generate wind power *Patv*^*i*^ separately, and the outcome power of the wind farm is the sum of all the wind turbines. In other words, at time *t*, the output power of the wind farm is $P={\sum }_{i}\;Pat{v}^{i}$. An illustration of a wind farm’s turbine array is shown in Fig. 1(a).

Here we describe the turbine characteristics used to generate the SDWPF dataset. The data is derived from the SL1500/82 turbine type, produced by Sinovel Wind Group Co., Ltd. This turbine type utilizes reliable doubly-fed power generation technology, with a rotor diameter of 82 meters and a hub height of 70 meters. It features three blades, each measuring 40.25 meters in length. For further information on the SL1500 series turbines, please refer to the manufacturer’s official website^23^. More information about the turbine is also presented in Supplementary Section B.1 with Table S1.

Aside from the SCADA data, we also included the weather data such as relative humidity, wind speed, wind direction, etc. collected from the fifth generation of the European Centre for Medium-Range Weather Forecasts (ECMWF) Atmospheric Reanalyses of the global climate (ERA5)^24^. The decision to utilize meteorological data from ERA5 is driven by the need to isolate errors stemming from Numerical Weather Prediction (NWP) from those inherent to the WPF challenge. ERA5 seamlessly integrates past observations with contemporary numerical models, yielding a consistent dataset spanning extensive temporal horizons. Its objective is to emulate real atmospheric conditions for each respective time step to the fullest extent feasible. In contrast, in practical applications, the accessible data primarily originates from NWP models, which anticipate the atmospheric state for a specific location and future time, leveraging both current and historical observational data. Although contemporary NWP models exhibit increasing precision, they inherently carry uncertainties, which become more pronounced for extended forecast durations. Using a reanalysis dataset such as ERA5 helps to mitigate the cumulative error inherent in NWP models, effectively decoupling the wind power forecasting problem from the challenges of weather prediction.

The relative position of all wind turbines in the wind turbine array is released to characterize the spatial correlation among wind turbines. An illustration of the spatial distribution of the total 134 wind turbines is shown in Fig. 1(b). All turbines are of the same type and have identical hub heights (defined as the distance from the turbine platform to the rotor, excluding the length of the blades^25^). The units of *x* and *y* are meters. In addition to the relative position, the elevation of each wind turbine is also provided in the dataset based on the Terra Advanced Spaceborne Thermal Emission and Reflection Radiometer (ASTER) Global Digital Elevation Model (GDEM) Version 3 (ASTGTM)^26^.

## Data Records

The SDWPF dataset spans from January 2020 to December 2021. It comprises SCADA data collected every 10 minutes from each of the 134 wind turbines in the wind farm. The 10-minute record data represents average values over each 10-minute interval, derived from high-frequency (1 Hz) sampling by the SCADA system. The SDWPF dataset can be accessed through the Figshare repository^27^. The key statistics and details of the SDWPF dataset are provided in Table 1.

**Table 1:** Statistics of the SDWPF data.

Time range	Interval	# of columns	# of turbines	# of records
Jan. 2020 to Dec. 2021	10 mins	19	134	11,361,191

An introduction to the main attributes of the data in Table 2. The dataset includes critical external features, e.g., wind speed, wind direction, and external temperature, as well as essential internal features, e.g., the inside temperature, nacelle direction, and pitch angle of blades. The external features influence the wind power generation, while the internal features can indicate the operating status of each wind turbine.

**Table 2:** Column names and their specifications of the SDWPF data.

Column	Column Name	Specification	Note
1	TurbID	Wind turbine ID	
2	Day	Day of the record	
3	Tmstamp	Created time of the record	Time zone UTC + 08:00
4	Wspd (m/s)	The wind speed at the top of the turbine	Recorded by mechanical anemometer
5	Wdir(°)	Relative wind direction, which is the angle between the wind direction and the the turbine nacelle direction	Wind direction and nacelle direction are in degrees from true north
6	Etmp (°C)	Temperature of the surrounding environment	Measured outer surface of the nacelle
7	Itmp (°C)	Temperature inside the turbine nacelle	
8	Ndir (°)	Nacelle direction, the yaw angle of the nacelle	In degree from true north
9	Pab1 (°)	Pitch angle of blade 1	The angle between the chord line and the rotation plane of the blade
10	Pab2 (°)	Pitch angle of blade 2	Same as above
11	Pab3 (°)	Pitch angle of blade 3	Same as above
12	Prtv (kW)	Reactive power	
13	T2m (°C)	Temperature at 2 m above surface (ERA5)	
14	Sp (Pa)	Surface pressure from ERA5	
15	RelH	Relative humidity	Derived based on 2 m dew point temperature and 2m temperature using Python Package metpy
16	Wspd_w (m/s)	Wind speed from ERA5	At height of 10 m
17	Wdir_w (°)	Wind direction from ERA5	At height of 10 m
18	Tp (m)	Total precipitation from ERA5	
19	Patv (kW)	Active power, the wind power produced by a wind turbine at a time stamp.	

We have released the dataset on the Figshare repository^27^. For easier utilization, we have divided the dataset into two parts: ***sdwpf_kddcup*** and ***sdwpf_full***. The *sdwpf_kddcup* comprises the original dataset used for the Baidu KDD Cup 2022, including both training and test datasets. The *sdwpf_full* provides a more extensive collection, featuring additional data not previously available during the KDD Cup, such as weather conditions, dates, and elevation. The *sdwpf_full* dataset contains three files, where *sdwpf_turb_location_elevation.csv* details the relative positions and elevations of all wind turbines within the dataset; *sdwpf_2001_2112_full.csv* includes data collected two years from the wind farm containing 134 wind turbines, spanning from Jan. 2020 to Dec. 2021; and *sdwpf_2001_2112_full.parquet* is identical to *sdwpf_2001_2112_full.csv*, but in a different data format. *sdwpf_full* offers comprehensive enhancements over the *sdwpf_kddcup* including extend time span, enriched weather information, and expanded temporal details. A detailed comparison between *sdwpf_full* and *sdwpf_kddcup* can be found in Supplementary Section B.2.

## Technical Validation

In this section, we begin with an ablation study to examine the features of the SDWPF dataset. Additionally, we discuss some of the results from the KDD Cup 2022, which is recognized as one of the most prestigious competitions in data science. More information about the experimental setting and evaluation experiment on the dataset can be found in Supplementary Section C.

### Ablation study of features

**Fig. 2:**
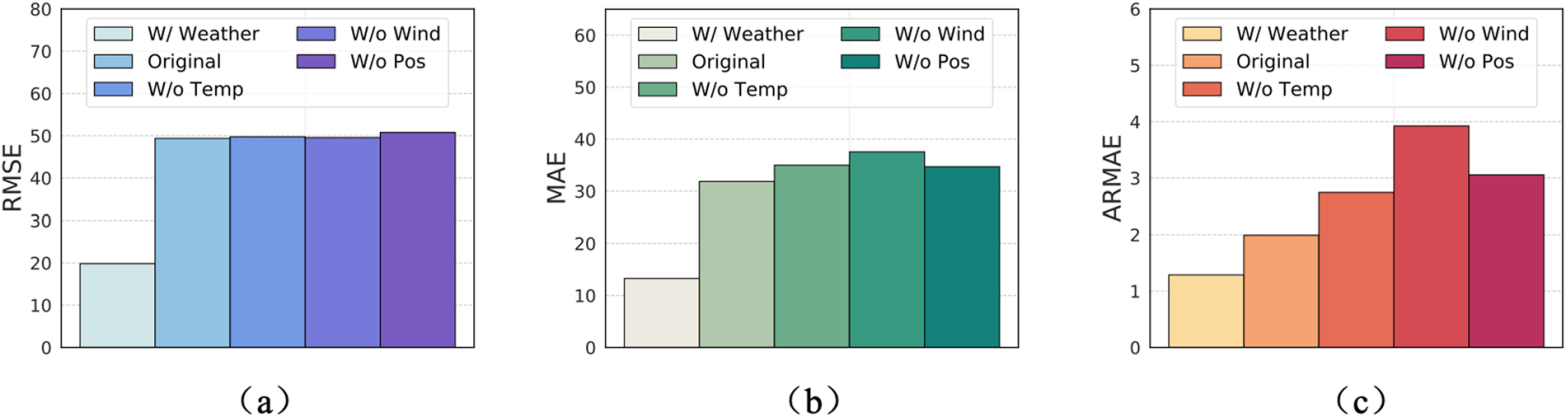
Ablation study of variables (weather forecast and dynamic context features) in the SDWPF dataset w.r.t. the wind power forecasting performance of the Informer model.

We conducted an ablation study to show the effectiveness of the additional features of SDWPF which is shown in Fig. 2. Here we use the Informer model to demonstrate this ablation study. **W/Weather** means to include the weather forecast features (in the future 48 days) that relate to the surrounding environment of the wind farm collected from ERA5 into the prediction model. **W/o Wind** means to remove the historical features of Wspd and Wdir from the input, **W/o Temp** means to remove the historical features of Etmp and Itmp from the input, and **W/o Pos** means to remove the historical features of Ndir, Pab1, Pab2, and Pab3 from the input.

At first, this study showcases the effectiveness of weather forecast information for WPF. As we can see from Fig. 2, if putting the weather forecast data into the model, the RMSE of the Informer can significantly decline from 49.368 to 19.828; and its MAE decline from 31.862 to 13.268. This assessment reveals that weather forecast data can substantially enhance the accuracy of WPF predictions.

Second, the ablation study also verifies that the dynamic context features are helpful for WPF. The MAE of Informer based on historical dynamic context features is 31.862. If removing the Temp context features, the MAE becomes 35.004; if removing the Wind context feature, the MAE becomes 37.562; and if removing the Pos context features, the MAE becomes 34.684. This demonstrates shows the effectiveness of adding the dynamic context features into the model for accuracy improvement.

### Evaluation on KDD Cup 2022

In the KDD Cup 2022 challenge, an evaluation score that considers both MAE and RMSE has been adopted to assess the performance of all participating teams. The specific evaluation setting details are in Supplementary Section C.4. It should be noted that the selection of an evaluation metric can differentially affect the rank of the competing methods utilized by the teams. For instance, the RMSE places more emphasis on large errors, resulting in a substantial penalty in situations where such errors are undesirable. To strike a balance between penalizing large errors and minimizing errors, we utilize the average of MAE and RMSE to assess the participating teams in the challenge.

**Fig. 3:**
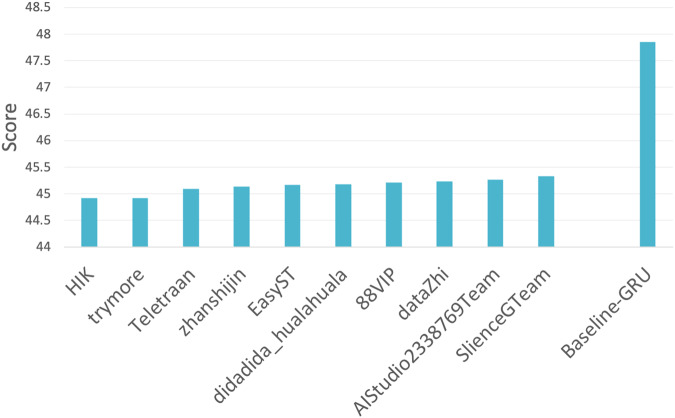
Performance evaluation on KDD Cup 2022. The X-axis shows the top ten participating teams in KDD Cup 2022 as well as the GRU baseline method. The evaluation score is a negatively oriented metric, signifying that lower values indicate better performance.

Figure 3 presents the evaluation scores of the top ten teams that participated in the KDD Cup 2022 challenge. Additionally, we compared all teams to a native baseline, namely the GRU. Note that the setting of Baidu KDD Cup 2022 is to forecast wind power solely based on historical information. Notably, we observed a substantial difference in prediction performance between the participating teams and the GRU baseline. The best evaluation score obtained by a team (HIK) was 44.917, where the baseline GRU score was 47.850 (as shown in Fig. 3). Therefore, the top-performing team reduced the prediction error (in terms of score in this setting) from the baseline GRU by 6.130%. It is worth noting that the improvement in evaluation score from the 10th team (SlienceGTeam) to the 1st team (HIK) was only 0.906% (SlienceGTeam vs. HIK: 45.327 vs. 44.917). This result highlights the high level of competitiveness in the KDD Cup challenge. Most of the top-performing teams have made their code open-source and published their technical reports on the official challenge website^22^. Thus, we expect to be able to evaluate future methods against these top-performing methods that deposited predictions in the Baidu KDD Cup 2022 challenge and therefore monitor the progress of wind power forecasting over time.

## Usage Notes

We introduce a few caveats about when to use this data to train and evaluate the models. Attention needs to be paid to these caveats since there are always some outliers and missing values in the data due to data collection, system maintenance, and equipment failures. It is important to note that we did not apply any of the following described corrections to the released dataset. The processing method introduced is only a suggestion, and the actual application of these methods is up to the dataset users.

### Zero values

For a wind turbine, some active and reactive power readings might be noted as slightly negative values. This phenomenon is often associated with specific components like the control system and sensors, which draw power even when the turbine is not producing electricity. We can treat all the values which are smaller than 0 as 0, i.e., if *Patv* < 0, then *Patv* = 0.

### Missing values

Note that due to some reasons, such as system maintenance and equipment failures, some sensor values at some time of a turbine are not collected from the SCADA system. These missing values will not be used for evaluating the prediction model. In other word, when $Pat{v}_{{t}_{0}+j}$ is a missing value, we set $\left\Vert Pat{v}_{{t}_{0}+j}-{\overline{Patv}}_{{t}_{0}+j}\right\Vert =0$ regardless of the actual predicted value of ${\overline{Patv}}_{{t}_{0}+j}$.

### Unknown values

Sometimes, the wind turbines are stopped from generating power by external reasons such as wind turbine renovation and/or actively scheduling the powering to avoid overloading the grid. In these cases, the actual generated power of the wind turbine is unknown. These unknown values should also not be used for evaluating the prediction model. Similarly with the missing values, if $Pat{v}_{{t}_{0}+j}$ is an unknown value, we always set $\left\Vert Pat{v}_{{t}_{0}+j}-{\overline{Patv}}_{{t}_{0}+j}\right\Vert =0$. Here we introduce two conditions to determine whether the target variable is unknown:

- When *Patv*≤0, and *Wspd* > 2.5 at time *t*, the actual active power *Patv* of this wind turbine at time *t* is unknown (since the wind speed is large enough to generate the power, the only reason that *Patv*≤0 is this turbine is stopped);
- When *Pab*1 > 89° or *Pab*2 > 89° or *Pab*3 > 89° (*Pab*1, *Pab*2, and *Pab*3 always have the same values) at time *t*, the actual active power *Patv* of this wind turbine at time *t* should be unknown (since no matter at then how large the wind speed is, the wind turbine is at rest in this situation).

### Abnormal values

There are some abnormal values collected from the SCADA system. If a data record has an abnormal value in any column, these values also should not be used for evaluating the model. Formally, if a wind turbine has an abnormal value at time *t*_0_ + *j* in any column, we always set $\left\Vert Pat{v}_{{t}_{0}+j}-{\overline{Patv}}_{{t}_{0}+j}\right\Vert =0$. Here we define two rules to identify abnormal values:

- The reasonable range for Ndir is [−720°, 720°], as the turbine system allows the nacelle to turn at most two rounds in one direction and would force the nacelle to return to the original position otherwise. Therefore, records beyond the range can be seen as outliers caused by the recording system. Thus, if at time *t*, there are Nidir > 720° or Nidir < −720°, then the recorded values of this wind turbine at time *t* is abnormal.
- The reasonable range for Wdir is [−180°, 180°]. Records beyond this range can be seen as outliers caused by the recording system. When there are Widr > 180° or Widr < −180° at time *t*, then the recorded values of this wind turbine at time *t* is abnormal.

In Table 3, we present statistics regarding the number of zero values, missing values, unknown values, and abnormal values (in the *sdwpf_full* data file). It is important to note that there is a significant overlap between zero values and unknown values. This overlap often arises because both can result from intentional adjustments, such as power scheduling to prevent grid overloading.

**Table 3:** The statistics of zero values, missing values, unknown values, and abnormal values in the *sdwpf_full* data file.

Total	Zero values	Missing values	Unknown values	Abnormal values
11,361,191	3,263,171	496,998	2,569,331	141

### Supplementary information

Supplementary of the manuscript

## Data Availability

The code to process the data and run baselines can be found in: https://github.com/PaddlePaddle/PaddleSpatial/tree/main/apps/wpf_baseline_gru.
