# Supplementary material for: SDWPF: A Dataset for Spatial Dynamic Wind Power Forecasting over a Large Turbine Array
Source: Sci Data. 2024 Jun 19;11:649. doi: 10.1038/s41597-024-03427-5 (PMC11187227; doi:10.1038/s41597-024-03427-5)
Supplement: Supplementary file 1 — Supplementary of the manuscript [file 41597_2024_3427_MOESM1_ESM.pdf]

SDWPF: A Dataset for Spatial Dynamic Wind Power Forecasting over a Large Turbine Array

Jingbo Zhou[1,*,†], Xinjiang Lu[1,†], Yixiong Xiao[1,†], Jian Tang[2,4,†], Jiantao Su[2,†], Yu Li[1], Ji Liu[3], Junfu Lyu[4], Yanjun Ma[3], Dejing Dou[5,6]

[1] Business Intelligence Lab, Baidu Research, Beijing, China

[2] China Longyuan Power Group Corp. Ltd., Beijing, China

[3] Baidu Inc., Beijing, China [4] Tsinghua University, Beijing, China

[5] BEDI Cloud, Beijing, China [6] Fudan University, Shanghai, China

* Contact email: zhoujingbo@baidu.com. †Equal contribution.

Supplementary

A Related Work

Wind power forecasting (WPF) has been extensively investigated over the past decade [1–5]. According to the spatial scale of the wind power, the problem can be categorized as the prediction of a single wind turbine, a wind farm, and a group of wind farms [6]. The SDWPF dataset belongs to the wind farm scale. The time scale to forecast the wind power also ranges from very short-term (in the range of a few minutes) to long-term (in the range of one day to one month) [7]. There are also some sub-tasks for WPF, e.g., ramp forecasting [8] and variability forecasting [9]. The former task aims to predict large and sharp variations in wind power. The latter aims to predict the large amplitude and periodic changes in wind power. The main difference between these tasks is not the methods but the metrics to evaluate the prediction quality.

Multiple delicate models have been specially designed for WPF problems with a variant of spatial and temporal scales based on statistic models [3, 10–12], machine learning methods [13–15] and deep learning methods [16–18]. Some advanced time series prediction methods [19–22] also have great potential to tackle this problem. A comprehensive review of seminal work on wind power forecasting is presented in [23, 24], while a survey focusing on recent advanced solutions in wind power forecasting can be found in [5, 25]. Several benchmarks and competitions for wind power forecasting exist, such as the wind forecasting track in the Global Energy Forecasting Competition [26, 27], as well as the recent initiatives by IEA Wind Task 51 Forecasting [28].

There are a few recent works to review the datasets for the WPF problem. Meneze et. al. examine a broader range of wind resource and wind farm datasets [29]. A study in [30] provides an overview of wind resource datasets but does not include turbine level datasets. Another study [31] divides the wind power datasets into three groups, including turbine-level SCADA datasets, turbine-level non-SCADA datasets, and aggregated datasets. The SDWPF dataset belongs to the first category, which contains the most useful information, e.g., environmental, operational, thermal, and electrical measurements obtained from SCADA system [32]. Whereas, the turbine-level non-SCADA datasets have only a limited amount of weather variables and do not have other dynamic contextual factors of a turbine. Aggregated data is aggregated over different regions, ranging from wind farms to whole countries. While being generally publicly available, aggregated data does not contain any turbine-level measurements or turbine-specific power output. For example, as a comprehensive archive for time series [33], it contains two wind farm datasets: the one is the Wind Farms Dataset which has long minutely wind power production time series of 339 wind farms in Australia, and the other one is the Wind Power Dataset which is a single very long time series of an Australian wind farm recorded per every 4 seconds. Both of them are extracted from the Australian Energy Market Operator (AEMO) online platform [34]. Another repository of wind farm datasets is housed within the Task 51 benchmark of the IEA Wind TCP [5]. This initiative was established to curate and disseminate benchmark test cases and data sets tailored for forecasting weather-driven energy systems. The archive compiles observed wind generation forecasts from four to seven wind farms situated in various countries globally (refer to https://iea-wind.org/task51/task51-information-portal/benchmarks/).

There are only a few public turbine-level SCADA datasets which can be comparable with the SDWPF dataset. According to a recent comprehensive survey [31], there are only 11 publicly available wind power datasets over the world that contain SCADA data. Three of them do not provide relative coordinates of a turbine, four of them have only 1 turbine, and seven of them have less than 10 turbines. For example, the Kelmarsh dataset has a turbine array with only 6 turbines [35], and the La Haute Borne dataset has only 4 turbines [36]. Except for SDWPF, the wind power dataset with the largest turbine array size is presented in [37] with 32 turbines (in contrast, SDWPF has 134 turbines). Besides, the dataset in [37] is not widely used due to the lack of an easy-accessible English document (which is originally presented in Portuguese) for the analysis of the dataset.

B Data Records

B.1 Turbine Characteristics

The wind farm is equipped with the Sinovel SL1500/82 turbine type, manufactured by Sinovel Wind Group Co., Ltd. Known for its reliable doubly-fed power generation technology, this turbine is part of the SL1500 series. Detailed characteristics of these turbines are available on the manufacturer's official website [38]. For your convenience, the key technical specifications of the turbine are detailed in Table S1.

Springer Nature 2021 LaTeX template

Table S1: Technical characteristics of the Sinovel SL1500/82 turbine type.

Turbine Parameter		Value	Turbine Parameter		Value
Operation	Rated power	1500 kw	Rotor	Blade number	3
	Cut-in wind speed	3 m/s		Rotor diameter	82 m
	Cut-out wind speed	25 m/s		Blade length	40.25 m
	Rated wind speed	10.5 m/s		Swept area of rotor	5938 m^2
	Average wind speed	8.5 m/s	Gearbox	Structural form	two-stage planetary and one-stage parallel shaft gears
	Survival wind speed	59.5 m/s			
	Operating temperature	[-15°C, 45°C]			
	Survival temperature	[-25°C, 50°C]	Yaw	Model	active
Generator	Model	doubly-fed, water-cooled	Tower	Model	steel conical tower
	Rated output voltage	690 v		Hub height	70 m
	Frequency	50 hz	Pitch	Control	motor + gearbox
	Rated speed	1800 rpm	Baking	Air brake	individual blade control
	Power factor	capacitive 0.9, inductive 0.9		Mechanical brake	hydraulic disc brake

B.2 Comparison between sdwpf_kddcup and sdwpf_full

We have released the dataset on the Figshare repository [39]. For easier utilization, we have divided the dataset into two parts: **sdwpf_kddcup** and **sdwpf_full**. The sdwpf_kddcup comprises the original dataset used for the Baidu KDD Cup 2022, including both training and test datasets. The sdwpf_full provides a more extensive collection, featuring additional data not previously available during the KDD Cup, such as weather conditions, dates, and elevation.

The **sdwpf_kddcup** dataset is the original dataset used for Baidu KDD Cup 2022 Challenge. The descriptions of each sub-folder in the sdwpf_kddcup dataset are as follows:

- *sdwpf_245days_v1.csv*: This dataset, released for the KDD Cup 2022 challenge, includes data spanning 245 days.
- *sdwpf_baidukddcup2022_turb_location.csv*: This file provides the relative positions of all wind turbines within the dataset.

- *final_phase_test*: This dataset serves as the test data for the final phase of the Baidu KDD Cup. It allows for a comparison of methodologies against those of the award-winning teams from KDD Cup 2022.

More information about the sdwpf_kddcup used for Baidu KDD Cup 2022 can be found in our technical report prepared for KDD Cup [40].

The **sdwpf_full** dataset offers more information than what was released for the KDD Cup 2022. It includes not only SCADA data but also weather data such as relative humidity, wind speed, and wind direction, sourced from the Fifth Generation of the European Centre for Medium-Range Weather Forecasts (ECMWF) atmospheric reanalyses of the global climate (ERA5). The dataset encompasses data collected over two years from a wind farm with 134 wind turbines, covering the period from January 2020 to December 2021. The descriptions of each sub-folder in the sdwpf_full dataset are as follows:

- *sdwpf_turb_location_elevation.csv* This file details the relative positions and elevations of all wind turbines within the dataset.
- *sdwpf_2001_2112_full.csv*: This dataset includes data collected two years from a wind farm containing 134 wind turbines, spanning from Jan. 2020 to Dec. 2021. It offers comprehensive enhancements over the sdwpf_kddcup/sdwpf_245days_v1.csv, including:
  - Extended time span: It encompasses two years' data from Jan. 2020 to Dec. 2021, whereas sdwpf_245days_v1.csv covers only 245 days.
  - Enriched weather information: This includes additional data such as relative humidity, wind speed, and wind direction, sourced from the Fifth generation of the European Centre for Medium-Range Weather Forecasts (ECMWF) atmospheric reanalyses of the global climate (ERA5).
  - Expanded temporal details: Unlike during the KDD Cup Challenge where timestamp information was withheld to prevent data linkage, this version includes specific timestamps for each data point.
- *sdwpf_2001_2112_full.parquet*: This dataset is identical to sdwpf_2001_2112_full.csv, but in a different data format.

B.3 Correlation Analysis

We conduct an exploratory analysis of the SDWPF dataset. We will briefly discuss temporal correlation (See Figure S1), context variables (see Figure S2), and spatial correlation (see Figure S3), respectively.

Temporal auto-correlation

Figure S1 presents the mean temporal auto-correlation of active power (Patv) of the turbine array. The auto-correlation for each turbine is computed for every time interval, and the results are subsequently averaged over all 134 turbines. As shown in Figure S1, the wind power values at different time points are correlated. The existence of temporal autocorrelation reveals the possibility of forecasting wind power data based

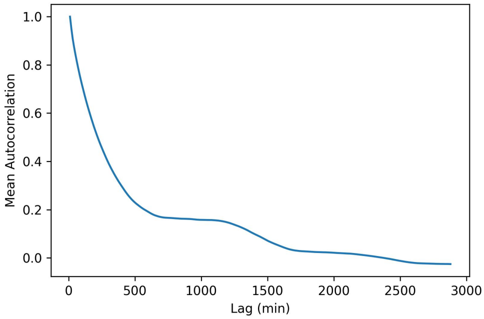

Fig. S1: Temporal auto-correlation of active power (i.e. Patv) over the wind turbine array.

on historical values. The autocorrelation approaches zero when the temporal lag is larger than two days.

Wind power and feature correlation

We use the paired correlation between turbines to show the relation between wind power and other dynamic context features. Taking Figure S2 (a) as an example, each point in the figure denotes a pair of turbines. The Y-axis denotes the Pearson correlation value between these two turbines under active power, and X-axis denotes the Pearson correlation value between these two turbines under a given feature. The intuition behind this figure is that, if a context feature (e.g. Wspd) can determine the active power with high possibility, then the high (or low) correlation between turbines under this context variable can result in a high (or low) correlation between them under the active power. As shown in Figure S2, wind power has a strong correlation between Wspd, Wdir, and pitch angles. We only show the result of Pab1 since Pab2 and Pab3 are almost the same as Pab1. However, the relations between wind power and other features are complex to interpret, such as Itmp, Ndir, and Etmp.

Wind power and spatial distribution correlation

As shown in Figure S3, we can see that there is a significant correlation between the wind power of a turbine and its spatial distribution. The complex relationship between the distance and power correlation is visualized in Figure S3 (a), where each point denotes a pair of turbines, the x-axis is the distance between the two turbines, and the y-axis is the Pearson correlation coefficient of the produced wind power between turbines. As shown in Figure S3 (a), in general, nearby wind turbines have a high correlation, but some nearby wind turbines have a low correlation, and some far away turbines have a high correlation. Figure S3 (b) shows that the elevation also has an impact on the wind power.

The correlation among turbines is determined by multiple factors. Except for the distance, another important factor is the wake effect: there will be a wake behind the turbine. The wake not only reduces wind speed but also increases the turbulence of

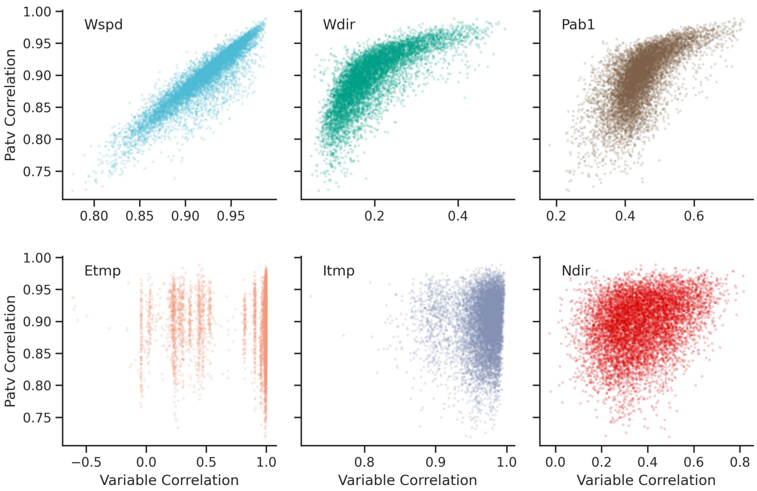

Fig. S2: The correlation distribution between wind power and ten context feature variables from our dataset. Each data point represents a pair of turbines. On the Y-axis, the correlation between these turbines in terms of active power is illustrated, while the X-axis indicates the correlation between the same turbines based on specified context feature variables. Taking the left-right subfigure pertaining to Wspd as an example, the X-axis reveals the correlation between the turbines concerning the wind speed as recorded by the anemometer (i.e., Wspd in Table 2). Conversely, the Y-axis demonstrates the correlation between the turbines in terms of active power. The presented figures elucidate the correlation distribution between active power and various context features.

the wind, both of which would disturb the wind power generation for the downwind turbines [41]. Our dataset, characterized by such a large turbine array, also presents a possible opportunity for investigating the wake effects within wind farms to enhance forecasting accuracy.

Both data analysis in Figure S3 and the physical modeling demonstrate that the spatial distribution (relative position and elevation) among turbines is important for WPF, but how to utilize such information by models is still an open problem. Existing time series prediction models cannot directly utilize such spatial information. During the Baidu KDD Cup 2022, we find that some awarded teams have verified the positive impact of this information on wind power forecasting. For example, Lin et al. [42] conduct an ablation study to show that, clustering the turbine according to the spatial location can significantly perform better than clustering the turbine according to the statistical correlation (See Section 3.4.1 and Figure 4 of [42]). There are at least three teams out of the top-10 awarded teams who have reported that the relative location information is useful for the WPF problem, whose solutions can be found in [42–44]. How to utilize such spatial distribution information in a novel model is beyond the scope of this paper, and we leave it as an interesting future research direction.

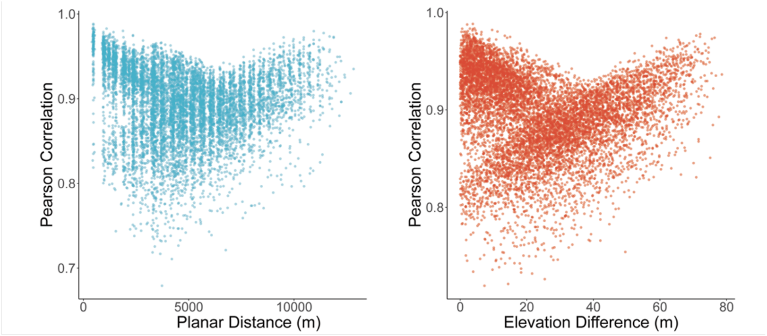

(a) (b)

Fig. S3: The correlation of the wind power and the spatial distribution between turbines. (a) Visualization of the relationships between the distance and wind power correlation. (b) Visualization of the relationship between elevation differences and wind power correlation. In (a) and (b), each point indicates a pair of turbines, the x-axis is the planar distance (a) and the difference in elevation (b) between these two turbines while the y-axis is the Pearson correlation coefficient.

C Experimental Evaluation

Here we present an experimental evaluation of the SDWPF dataset on several baselines. In our experiment, we build time series prediction models to predict the active power for each wind turbine. We build several state-of-the-art time series prediction models, include GRU [45], LightGBM [46], N-BEATS [47], DeepAR [48], Informer [49], Autoformer [50] and Reformer [51]. We also introduce a simple baseline based on the statistical model SARIMAX which is an updated version of the ARIMA [52, 53]. Then we use the data of the 214 days starting from May 1. 2020 as a training dataset, the next 16 days as a validation dataset, and the following 15 days as a testing dataset. For SARIMAX, in every step we use the past seven days' data to fit the model and then forecast the value of the next 48 hours for every turbine. The hyperparameter setting can be found in Supplementary Section C.2. Experiments are conducted on a GPU-CPU platform. The GPU is NVIDIA A100, and the CPU is Intel(R) Xeon(R) Gold 6271C. We use the metrics of RMSE (Root Mean Square Error), MAE (Mean Absolute Error), and ARMAE (Accumulative Relative Mean Absolute Error) to evaluate the performance of baseline models on our dataset. An introduction to the evaluation metrics can be found in Supplementary Section C.1. We also present the power curves of three turbines, demonstrating the variability in output active power across different wind speeds for different turbines in Supplementary Section C.8.

C.1 Evaluation Metrics

We evaluate the prediction results for each wind turbine, and the sum of the prediction scores can be the final score of the wind farm. The evaluation metrics $RMSE^i_{t_0}$ and $MAE^i_{t_0}$ for wind turbine i at the time step t_0 for h step ahead prediction is defined as:

$$rmse_{t_0}^i = \sqrt{\frac{\sum_{j=1}^{h}(Patv_{t_0+j}^i - \overline{Patv}_{t_0+j}^i)^2}{h}},\ mae_{t_0}^i = \frac{\sum_{j=1}^{h}\|Patv_{t_0+j}^i - \overline{Patv}_{t_0+j}^i\|}{h} \tag{1}$$

where $Patv_{t_0+j}^i$ is the actual power of wind turbine i and $\overline{Patv}_{t_0+j}^i$ is the predicted power of the wind turbine i at time step $t_0 + j$. Note that each time step of j is 10 minutes, thus there are 288 steps for 48 hours ahead prediction. The overall score of the wind farm of the prediction model is the sum of the metrics on all wind turbines, i.e.:

$$RMSE_{t_0} = \sum_{i=1}^{134} rmse_{t_0}^i,\ \ MAE_{t_0} = \sum_{i=1}^{134} mae_{t_0}^i \tag{2}$$

We also define an Accumulative Relative Mean Absolute Error (ARMAE), which is computed by:

$$ARMAE_{t_0} = \frac{MAE_{t_0}}{\sum_{j=1}^{h}\|Patv_{t_0+j}^i\|/h} \tag{3}$$

A length-L_x-length-h prediction window is adopted to roll the whole test set with stride Δt time steps (Each time step of Δt is 10 minutes), and the averaged evaluation score is reported. Note that, L_x denotes the length of input time series. In our evaluation, we set the maximum length of the input time series L_x as 14 days. In the experimental evaluation, we use $\mathbf{K}$ instances to evaluate the performance of the prediction model:

$$RMSE = \frac{1}{K}\sum_{k=0}^{\mathbf{K}} RMSE_{t_0+k*\Delta t_r}, \tag{4}$$

$$MAE = \frac{1}{K}\sum_{k=0}^{\mathbf{K}} MAE_{t_0+k*\Delta t_r} \tag{5}$$

$$ARMAE = \frac{1}{K}\sum_{k=0}^{\mathbf{K}} ARMAE_{t_0+k*\Delta t_r} \tag{6}$$

C.2 Hyper-Parameter Settings

The hyper-parameters are selected on the validation dataset. For all the deep methods (i.e., GRU, Informer, Autoformer, Reformer, DeepAR, and N-Beats), the batch size is set as 16 and the dimension of the embedding is set as 512. The size of hidden states and the layer of the recurrent network are set as 24 and 2 for GRU, and 32 and 1 for DeepAR, respectively. For all the Transformer-based methods, the head number of multi-head attention is set as 8, the layer of the encoder is set as 2 and the layer of the decoder is set as 1. And the length of the encoder's input sequence is 288, and the

**Baselines**	RMSE	MAE	ARMAE
GRU	54.27	45.981	5.545
Informer	49.368	31.862	1.991
Autoformer	52.618	39.358	3.494
Reformer	51.840	43.678	5.191
N-BEATS	86.725	82.989	10.740
DeepAR	53.011	43.660	4.586
LightGBM	50.585	47.387	1.839
SARIMAX	104.865	83.467	11.186

Table S2: Overall performance comparisons of different baseline methods on the SDWPF dataset for 48 hours ahead for wind power forecasting.

length of the start token of the decoder is 144. For Autoformer, the number of steps of moving average operation is 25. And for Reformer, the bucket length is set as 24. Besides, for N-Beats, 3 trend blocks and 3 seasonality blocks are adopted, and both the layer of trend block and seasonality block is set as 4, and the width of each layer is set as 512.

C.3 Overall Prediction Performance

We show the overall performance comparisons of different baseline methods in Table S2. In the evaluation, we conduct the 48 hours ahead wind power forecasting, and the overall prediction performance is shown in Table S3. In general, the Informer can achieve the best prediction performance over all baselines on the SDWPF dataset. It is worth noting that the LightGBM has the second-best performance under the RMSE. However, all the baselines have relatively large values under the ARMAE, which indicates the difficulty of making the WPF.

In Supplementary Table S3, we divide the 48 hours into eight time intervals (each interval is 6 hours) to show the prediction performance at different time scales. We also evaluate the deep learning baselines with a large rolling step ($\Delta t = 4\ hours$) in Table S4 which can be faster but without losing the robustness of the results. We also conducted an efficiency evaluation of all the baselines in Supplementary Section C.7 which shows all the baselines can make predictions in real-time.

C.4 Setting for KDD Cup 2022

In this section, we offer a brief overview of the Baidu KDD Cup 2022 challenge. Models are evaluated using a privately held dataset to ascertain the rankings of the participating teams.

C.4.1 Prediction Task

The prediction task of SDWPF is to predict the wind power of each turbine in advance. In KDD Cup 2022, we require the participants to address the prediction ahead of 48 hours. For example, given at 6:00 A.M. today, it is required to effectively forecast the

wind power generation beginning from 6:00 A.M. on this day to 5:50 AM on the day after tomorrow, given a series of historical records of the wind turbines and the related wind turbines. It is required to output the predicted values every 10 minutes. To be specific, at one time point, it is required to predict a future length-288 wind power supply time series for 48 hours ahead prediction. Though during the Baidu KDD Cup 2022 challenge, we aim to predict the wind power on top of the spatiotemporal modeling paradigm without knowing the future weather data (wind speed, temperature, etc.) of the wind farm, we have added the ERA5 weather data in the updated version of the data. The prediction task can take the ERA5 data as weather forecast data to enable different prediction modes.

C.4.2 Evaluation Setting

In the final phase of the Baidu KDD Cup 2022 challenge, we privately held data 143 instances randomly sampled from a half-year data (from 01/05/2021 to 10/31/2021) to evaluate the submitted model. The test dataset now is released on our website. The variability of wind power on test data may lead to unstable performance for the models. To alleviate the effect of such variability, we publicly release one-year data as a training dataset and use the data over the next half a year to evaluate the submitted models by participants. Formally, We use $\mathbf{K}$ data instances to evaluate the performance of the prediction model. For each data instance k, we randomly sample a stride time step Δt_k from the range [1, 10]. In other words, the stride time step randomly ranged from 10 minutes to 100 minutes. For the evaluation in our submission system, we use 143 random sampled stride time steps (i.e. $\{\Delta t_0, \Delta t_1, ..., \Delta t_{143}\}$) over the next several months to evaluate the submitted models. This test time range and sampled stride time steps will be updated in different competition phases. The details and the sample code about such an evaluation method can be found in the evaluation manual [1].

The score to rank the teams is defined as the average of RMSE and MAE, which are:

$$score = \frac{1}{2}(RMSE + MAE) \tag{7}$$

where the definition of $RMSE$ and MAE is the same with Equation 4 and Equation 5 in Supplementary Section C.1.

C.5 Performance Evaluation with Different Time Intervals

As shown in Table S3, we divide the 48 hours into eight time intervals (each interval is 6 hours) to show the prediction performance at different time scales. Especially, for the short-term forecasting with six hours (1st 00:00-05:50), the prediction accuracy of Informer is significantly better than its competitors, where the RMSE and MAE of Informer are 30.294 and 18.178 respectively, and the second best baseline is Reformer whose RMSE and MAE are 41.039 and 31.783. However, the superiority of Informer is degraded for the long-term prediction. The RMSE of Informer at the last six hours (2nd 18:00-23:50) is even worse than all its competitors. The MAE of Informer (i.e. 36.633) at the last six hours (2nd 18:00-23:50) is still better than its competitors.

[1] https://github.com/PaddlePaddle/PaddleSpatial/tree/main/apps/wpf_baseline_gru/kddcup22-sdwpf-evaluation

The reason leading to such difference in different metrics is that the RMSE metric gives a higher weight to large errors than the MAE metric, since for RMSE the errors are squared before they are averaged. These experimental results demonstrate the difficulty of making wind power forecasting, and the necessity to select a suitable model for conducting the prediction.

Table S3: Overall performance comparisons of different baseline methods on the SDWPF dataset for 48 hours ahead wind power forecasting. In the experiments, we denote 00:00 – 05:50 as night, 06:00 – 11:50 as morning (mor.), 12:00 – 17:50 as noon and 18:00 – 23:50 as evening (evn.).

Method		Time Period								
		1st Night	1st Mor.	1st Noon	1st Evn.	2nd Night	2nd Mor.	2nd Noon	2nd Evn.	Overall (48 h)
GRU	RMSE	46.479	48.982	53.004	53.735	53.547	53.186	53.472	54.279	54.279
	MAE	38.623	36.402	38.247	38.560	38.060	37.389	37.502	38.192	45.981
	ARMAE	5.398	6.247	6.318	5.922	5.543	5.159	4.934	4.838	5.545
Informer	RMSE	30.294	42.853	49.110	51.347	54.182	54.472	55.721	56.963	49.368
	MAE	18.178	27.348	32.567	33.713	34.701	35.178	36.578	36.633	31.862
	ARMAE	1.776	2.056	2.074	2.144	1.926	2.087	1.973	1.895	1.991
Autoformer	RMSE	40.109	53.197	55.727	55.143	54.249	53.620	53.816	55.081	52.618
	MAE	29.423	40.241	41.873	41.485	40.752	40.040	40.101	40.946	39.358
	ARMAE	2.766	3.789	3.625	3.622	3.645	3.541	3.493	3.474	3.494
Reformer	RMSE	41.039	48.941	50.686	52.243	54.222	55.328	56.018	56.242	51.840
	MAE	31.783	42.121	43.310	44.618	45.999	46.937	47.249	47.409	43.678
	ARMAE	3.778	5.392	5.216	5.349	5.490	5.542	5.422	5.340	5.191
N-BEATS	RMSE	87.698	87.661	86.879	86.652	86.328	86.165	86.364	86.056	86.725
	MAE	84.169	84.170	83.188	82.852	82.533	82.355	82.392	81.528	82.898
	ARMAE	11.349	11.189	10.781	10.783	10.744	10.527	10.372	10.176	10.740
DeepAR	RMSE	45.800	51.533	54.218	54.434	54.457	54.370	54.454	54.824	53.011
	MAE	36.192	42.342	44.871	45.165	45.205	45.083	45.078	45.343	43.660
	ARMAE	3.878	4.675	4.748	4.774	4.802	4.734	4.578	4.502	4.586
LightGBM	RMSE	51.343	52.946	46.114	41.718	57.325	59.445	50.726	45.0667	50.585
	MAE	48.934	51.312	41.071	38.188	54.955	57.629	45.464	41.543	47.387
	ARMAE	1.923	1.778	1.418	1.665	2.192	2.275	1.780	1.681	1.839
SARIMAX	RMSE	54.769	76.184	87.862	98.192	113.097	129.376	140.057	139.384	104.864
	MAE	38.599	56.845	67.359	77.291	91.498	106.193	115.332	114.628	83.467
	ARMAE	6.976	9.834	10.865	11.026	11.588	12.714	13.358	13.124	11.186

Since the efficiency of the deep learning-based models (GRU, Informer, Autoformer, Reformer, N-BEATS, and DeepAR) always have a concern, we also evaluate

our dataset with a large rolling step which can be faster but without losing the robustness of the results. Table S4 shows the prediction performance of these deep learning baselines with rolling step = 4 hours. As we can see, the prediction performance of the baselines is almost the same as the ones with rolling step = 10 minutes.

Table S4: Performance comparisons of different baseline methods on the SDWPF dataset with rolling step $\Delta t = 4$ hours.

Method		Time Period								
		1st Night	1st Mor.	1st Noon	1st Evn.	2nd Night	2nd Mor.	2nd Noon	2nd Evn.	Overall (48 h)
GRU	RMSE	46.288	49.102	51.209	51.985	51.599	51.921	51.381	52.454	50.742
	MAE	41.256	42.298	43.448	43.682	43.541	43.556	43.182	43.863	43.103
	ARMAE	5.275	4.794	4.833	4.491	4.787	4.525	4.461	4.355	4.690
Informer	RMSE	31.851	42.716	46.764	48.849	49.321	52.867	54.212	56.110	47.836
	MAE	19.756	31.149	33.548	33.963	33.179	35.375	33.526	33.629	31.766
	ARMAE	1.954	3.483	2.892	2.657	2.576	2.498	1.943	1.740	2.468
Autoformer	RMSE	38.692	48.934	52.050	52.969	52.803	53.680	54.218	57.054	51.300
	MAE	25.767	31.430	33.849	34.045	34.098	34.103	35.140	38.161	33.324
	ARMAE	2.435	2.069	2.223	1.933	2.262	2.089	2.464	2.644	2.265
Reformer	RMSE	46.640	51.182	53.383	54.027	53.810	54.086	53.644	54.463	52.654
	MAE	40.296	45.359	46.970	47.261	47.213	47.290	46.932	47.476	46.100
	ARMAE	5.273	5.282	5.433	5.068	5.412	5.141	5.058	4.956	5.203
N-BEATS	RMSE	87.430	88.018	86.766	86.922	86.032	86.580	86.226	86.713	83.048
	MAE	83.904	84.644	83.092	83.169	82.191	82.827	82.301	82.260	83.048
	ARMAE	11.797	11.131	11.068	10.490	10.996	10.568	10.403	10.283	10.842
DeepAR	RMSE	44.570	50.702	53.760	54.602	54.256	54.546	54.152	55.126	52.714
	MAE	35.661	41.837	44.641	45.213	45.105	45.177	44.902	45.614	43.519
	ARMAE	4.066	4.683	4.979	4.701	4.500	4.782	4.670	4.680	4.695

C.6 Ablation Study of Features

Table S5 shows the effect of different features with dividing the 48 hours into eight time intervals (each interval is 6 hours). We can see that results are generally consistent with Figure 8. An interesting point is that, after including the weather forecast data into the model, the prediction error for the relatively long term (after 24 hours) is not significantly larger than the one of the short-term prediction (within 24 hours). In other words, the prediction difficulty of the problem does not significantly increase when the length of the prediction time becomes large.

Table S5: Ablation study of different contexts in the SDWPF dataset w.r.t. the wind power forecasting performance of the Informer method. Wind context includes features of Wspd and Wdir, Temp context includes features of Etmp and Itmp, and Pos context includes features of Ndir, Pab1, Pab2 and Pab3. Besides, W/Weather means to include the forecast weather recordings that relate to the surrounding environment of the wind farm.

Method		Time Period								
		1st Night	1st Mor.	1st Noon	1st Evn.	2nd Night	2nd Mor.	2nd Noon	2nd Evn.	Overall (48 h)
w/ Weather	RMSE	21.091	17.905	18.199	18.004	17.991	19.912	21.783	23.743	19.828
	MAE	14.800	11.898	12.011	11.670	12.072	13.325	14.680	15.692	13.268
	ARMAE	2.339	1.144	1.141	0.923	1.096	1.149	1.360	1.141	1.286
Original	RMSE	31.851	42.716	46.764	48.849	49.321	52.867	54.212	56.110	47.836
	MAE	19.756	31.149	33.548	33.963	33.179	35.375	33.526	33.629	31.766
	ARMAE	1.954	3.483	2.892	2.657	2.576	2.498	1.943	1.740	2.468
w/o Wind	RMSE	35.412	46.267	53.329	53.648	51.348	53.079	50.875	52.576	49.567
	MAE	25.031	33.611	36.895	36.422	34.732	38.889	37.294	37.160	35.004
	ARMAE	2.986	2.604	2.288	2.286	2.847	2.976	3.000	2.972	2.745
w/o Tmp.	RMSE	33.349	45.060	47.515	52.465	56.370	55.888	56.398	59.188	50.779
	MAE	21.184	32.711	36.086	39.926	41.129	41.353	42.529	45.577	37.562
	ARMAE	2.174	3.385	3.179	3.855	4.307	4.298	4.780	5.401	3.923
w/o Pos.	RMSE	31.777	41.172	52.004	55.544	55.100	55.086	52.755	54.599	49.755
	MAE	21.382	30.744	39.762	40.252	38.307	37.819	33.500	35.709	34.684
	ARMAE	2.621	3.402	4.712	3.852	2.849	2.420	1.964	2.635	3.057

C.7 Efficiency

Table S6 shows the prediction time and training time per epoch in the SDWPF dataset. The prediction per step means to make a prediction for all the turbines (i.e. 134 turbines) per step. Note that since we need to re-fit the SARIMAX for every prediction step, SARIMAX does not have a large training time cost, but has a relatively large prediction time cost. We can see that these baselines can make predictions for all turbines in real time, which is practical for real-life applications.

C.8 Power curve and weather forecast data.

We also showcase the power curve of different turbines, indicating how large the output active power is for the turbine at diverse wind speeds. Special attention is paid to wind speed forecasting for wind power forecasting since generated wind power is proportional to wind speed cubed [54]. The generated wind power can be approximately estimated to be proportional to wind speed cubed [54]. The correlation between wind speed and output power is intricate. As illustrated in Figure S4, the power curve of certain turbines (as seen in the left panel of Figure S4) aligns closely

Table S6: Training time consumption of different baseline methods on the SDWPF dataset for 48 hours ahead wind power forecasting.

	Method	GRU	Informer	Autoformer	Reformer	N-Beats	DeepAR	LightGBM	SARIMAX
Time cost	Predict per step(ms)	221.94	432.15	560.29	402.00	249.56	505.85	512.28	4.74e4
	Train per epoch (s)	6.81e3	1.46e4	1.94e4	1.41e4	1.09e4	1.36e5	22.0	-

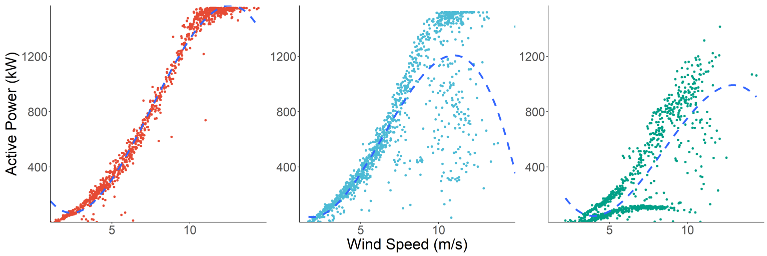

Fig. S4: The power curve of different turbines indicates how large the output active power is for the turbine at different wind speeds. The relationship between wind speed and wind power is complex and depends on the characteristics of different turbines.

with a cubic curve. Nonetheless, numerous turbines exist for which polynomial curves do not adequately model their power curves.

Figure S4 illustrates the complex relationship between wind speed and wind power, which varies significantly across different turbines. The difficulty of predicting wind power increases from left to right. For instance, the left subplot in Figure S4 suggested a distinct functional relationship between wind speed and wind power. Conversely, the power curve of the turbine shown in the middle one of Figure S4 is greatly distorted by noisy and outlier points. This indicated challenges in modeling this relationship due to numerous outliers, making predictions difficult. Moreover, the power curve of some turbines, like the one illustrated in the right part of Figure S4, even cannot be modeled by a single curve. The right subplot showed two different curves fitting the wind speed-power points, indicating that no unique mapping exists between wind speed and wind power.

The complexity of the relationship between wind speed and wind power is due to the varied behaviors exhibited by wind turbines under different conditions. For instance, wind turbines only generate power when wind speeds exceed a certain threshold. At extremely high wind speeds, turbines may operate at full capacity or even shut down to prevent damage. Additionally, factors such as local turbulence and complex terrain can significantly alter the shape of the wind power curve. Operational status, such as maintenance, and intrinsic characteristics of the turbines also play crucial roles. These elements indicate that turbine performance at the same wind

speed can be affected by multiple dynamic contextual factors, leading to the variability observed in power output.

It is widely considered a challenging problem for wind power forecasting without weather forecast data. If given weather forecast data, implicitly or explicitly modeling the relation between forecast weather information (like wind speed) and power may become the most important research problem. Both of the solutions to wind power forecasting, including those based on historical data and weather forecast data, should greatly benefit the WPF task.

References

[1] Wang, X., Guo, P., Huang, X.: A review of wind power forecasting models. Energy procedia **12**, 770–778 (2011)

[2] Foley, A.M., Leahy, P.G., Marvuglia, A., McKeogh, E.J.: Current methods and advances in forecasting of wind power generation. Renewable energy **37**(1), 1–8 (2012)

[3] Sideratos, G., Hatziargyriou, N.D.: An advanced statistical method for wind power forecasting. IEEE Transactions on power systems **22**(1), 258–265 (2007)

[4] Deng, X., Shao, H., Hu, C., Jiang, D., Jiang, Y.: Wind power forecasting methods based on deep learning: A survey. Computer Modeling in Engineering and Sciences **122**(1), 273 (2020)

[5] In: Möhrlen, C., Zack, J.W., Giebel, G. (eds.) IEA Wind Recommended Practice for the Implementation of Renewable Energy Forecasting Solutions. Wind Energy Engineering. Elsevier (2023)

[6] Jiang, Z.-Y., Jia, Q.-S., Guan, X.: A review of multi-temporal-and-spatial-scale wind power forecasting method. Acta Automatica Sinica **45**(1), 51–71 (2019)

[7] Hanifi, S., Liu, X., Lin, Z., Lotfian, S.: A critical review of wind power forecasting methods—past, present and future. Energies **13**(15), 3764 (2020)

[8] Gallego-Castillo, C., Cuerva-Tejero, A., Lopez-Garcia, O.: A review on the recent history of wind power ramp forecasting. Renewable and Sustainable Energy Reviews **52**, 1148–1157 (2015)

[9] Giebel, G., Kariniotakis, G.: Wind power forecasting—a review of the state of the art. Renewable energy forecasting, 59–109 (2017)

[10] Milligan, M., Schwartz, M., Wan, Y.-h.: Statistical wind power forecasting models: Results for us wind farms. Technical report, National Renewable Energy Lab.(NREL), Golden, CO (United States) (2003)

[11] Pinson, P., Madsen, H., Nielsen, H.A., Papaefthymiou, G., Klöckl, B.: From

probabilistic forecasts to statistical scenarios of short-term wind power production. Wind Energy: An International Journal for Progress and Applications in Wind Power Conversion Technology **12**(1), 51–62 (2009)

[12] Cassola, F., Burlando, M.: Wind speed and wind energy forecast through kalman filtering of numerical weather prediction model output. Applied energy **99**, 154–166 (2012)

[13] Kariniotakis, G., Stavrakakis, G., Nogaret, E.: Wind power forecasting using advanced neural networks models. IEEE transactions on Energy conversion **11**(4), 762–767 (1996)

[14] Zeng, J., Qiao, W.: Support vector machine-based short-term wind power forecasting, pp. 1–8 (2011). IEEE

[15] Hu, Q., Zhang, S., Yu, M., Xie, Z.: Short-term wind speed or power forecasting with heteroscedastic support vector regression. IEEE Transactions on Sustainable Energy **7**(1), 241–249 (2015)

[16] Wang, H.-z., Li, G.-q., Wang, G.-b., Peng, J.-c., Jiang, H., Liu, Y.-t.: Deep learning based ensemble approach for probabilistic wind power forecasting. Applied energy **188**, 56–70 (2017)

[17] Hong, Y.-Y., Rioflorido, C.L.P.P.: A hybrid deep learning-based neural network for 24-h ahead wind power forecasting. Applied Energy **250**, 530–539 (2019)

[18] Cui, W., Wan, C., Song, Y.: Ensemble deep learning-based non-crossing quantile regression for nonparametric probabilistic forecasting of wind power generation. IEEE Transactions on Power Systems (2022)

[19] Zhou, J., Tung, A.K.: Smiler: A semi-lazy time series prediction system for sensors. In: Proceedings of the 2015 ACM SIGMOD International Conference on Management of Data, pp. 1871–1886 (2015)

[20] Liang, Y., Ke, S., Zhang, J., Yi, X., Zheng, Y.: Geoman: Multi-level attention networks for geo-sensory time series prediction. In: Proceedings of the 27th International Joint Conference on Artificial Intelligence, vol. 2018, pp. 3428–3434 (2018)

[21] Hu, J., Zheng, W.: Multistage attention network for multivariate time series prediction. Neurocomputing **383**, 122–137 (2020)

[22] Li, T., Zhang, J., Bao, K., Liang, Y., Li, Y., Zheng, Y.: Autost: Efficient neural architecture search for spatio-temporal prediction. In: Proceedings of the 26th ACM SIGKDD International Conference on Knowledge Discovery & Data Mining, pp. 794–802 (2020)

[23] Giebel, G., Brownsword, R., Kariniotakis, G., Denhard, M., Draxl, C.: The state-of-the-art in short-term prediction of wind power: A literature overview (2011)

[24] Costa, A., Crespo, A., Navarro, J., Lizcano, G., Madsen, H., Feitosa, E.: A review on the young history of the wind power short-term prediction. Renewable and Sustainable Energy Reviews **12**(6), 1725–1744 (2008)

[25] Wang, Y., Zou, R., Liu, F., Zhang, L., Liu, Q.: A review of wind speed and wind power forecasting with deep neural networks. Applied Energy **304**, 117766 (2021)

[26] Hong, T., Pinson, P., Fan, S.: Global energy forecasting competition 2012. Elsevier (2014)

[27] Hong, T., Pinson, P., Fan, S., Zareipour, H., Troccoli, A., Hyndman, R.J.: Probabilistic energy forecasting: Global energy forecasting competition 2014 and beyond. Elsevier (2016)

[28] Giebel, G., Draxl, C., Frank, H., Zack, J., Möhrlen, C., Kariniotakis, G., Browell, J., Bessa, R., Lenaghan, D.: Iea wind task 51 forecasting for the weather driven energy system. In: EGU General Assembly 2023 (2023)

[29] Menezes, D., Mendes, M., Almeida, J.A., Farinha, T.: Wind farm and resource datasets: A comprehensive survey and overview. Energies **13**(18), 4702 (2020)

[30] Clifton, A., Hodge, B.-M., Draxl, C., Badger, J., Habte, A.: Wind and solar resource data sets. Wiley Interdisciplinary Reviews: Energy and Environment **7**(2), 276 (2018)

[31] Effenberger, N., Ludwig, N.: A collection and categorization of open-source wind and wind power datasets. Wind Energy **25**(10), 1659–1683 (2022)

[32] Astolfi, D., Castellani, F., Lombardi, A., Terzi, L.: Multivariate scada data analysis methods for real-world wind turbine power curve monitoring. Energies **14**(4), 1105 (2021)

[33] Godahewa, R.W., Bergmeir, C., Webb, G.I., Hyndman, R., Montero-Manso, P.: Monash time series forecasting archive. In: Thirty-fifth Conference on Neural Information Processing Systems Datasets and Benchmarks Track (2021)

[34] AEMO: Australian Energy Market Operator platform. https://aemo.com.au/. Online; accessed 29 August 2022 (2022)

[35] Kelmarsh wind farm data. https://zenodo.org/record/5841834#.YqEShRPP10s. Online; accessed June 06 2022 (2022)

[36] La haute borne wind farm data. https://opendata-renewables.engie.com/explore/

index. Online; accessed June 06 2022 (2022)

[37] Sakagami, Y., et al.: Influência da turbulência e do perfil de velocidade do vento no desempenho de aerogeradores em dois parques eólicos na costa no nordeste brasileiro (2017)

[38] Sinovel Wind Group Co., L.: SL1500 Series Wind Turbine. http://www.sinovel.com/english/content/?106.html. Online; accessed April 28 2024 (2024)

[39] Zhou, J., Lu, X., Xiao, Y., Tang, J., Su, J., Li, Y., Liu, J., Lyu, J., Ma, Y., Dou, D.: SDWPF: A Dataset for Spatial Dynamic Wind Power Forecasting over a Large Turbine Array (2023). https://doi.org/10.6084/m9.figshare.24798654. figshare

[40] Zhou, J., Lu, X., Xiao, Y., Su, J., Lyu, J., Ma, Y., Dou, D.: Sdwpf: A dataset for spatial dynamic wind power forecasting challenge at kdd cup 2022. arXiv preprint arXiv:2208.04360 (2022)

[41] González-Longatt, F., Wall, P., Terzija, V.: Wake effect in wind farm performance: Steady-state and dynamic behavior. Renewable Energy **39**(1), 329–338 (2012)

[42] Lin, F., Jiang, W., Zhang, H., Yang, C.: Kdd cup 2022 wind power forecasting team 88vip solution. Baidu KDD Cup 2022 (2022)

[43] Liu, H.: trymore: Solution to spatial dynamic wind power forecasting for kdd cup 2022. Baidu KDD Cup 2022 (2022)

[44] Kalander, M., Rao, Z., Zhang, C.: Wind power forecasting with deep learning: Team didadida_hualahuala. Baidu KDD Cup 2022 (2022)

[45] Cho, K., van Merriënboer, B., Bahdanau, D., Bengio, Y.: On the properties of neural machine translation: Encoder–decoder approaches. In: Proceedings of Eighth Workshop on Syntax, Semantics and Structure in Statistical Translation, pp. 103–111 (2014)

[46] Ke, G., Meng, Q., Finley, T., Wang, T., Chen, W., Ma, W., Ye, Q., Liu, T.-Y.: Lightgbm: A highly efficient gradient boosting decision tree. Advances in neural information processing systems **30** (2017)

[47] Oreshkin, B.N., Carpov, D., Chapados, N., Bengio, Y.: N-beats: Neural basis expansion analysis for interpretable time series forecasting. In: International Conference on Learning Representations (2020)

[48] Salinas, D., Flunkert, V., Gasthaus, J., Januschowski, T.: Deepar: Probabilistic forecasting with autoregressive recurrent networks. International Journal of Forecasting **36**(3), 1181–1191 (2020)

[49] Zhou, H., Zhang, S., Peng, J., Zhang, S., Li, J., Xiong, H., Zhang, W.: Informer: Beyond efficient transformer for long sequence time-series forecasting. In:

Proceedings of the AAAI Conference on Artificial Intelligence (2021)

[50] Xu, J., Wang, J., Long, M., et al.: Autoformer: Decomposition transformers with auto-correlation for long-term series forecasting. Advances in Neural Information Processing Systems **34** (2021)

[51] Kitaev, N., Kaiser, L., Levskaya, A.: Reformer: The efficient transformer. In: International Conference on Learning Representations (2019)

[52] Vagropoulos, S.I., Chouliaras, G., Kardakos, E.G., Simoglou, C.K., Bakirtzis, A.G.: Comparison of sarimax, sarima, modified sarima and ann-based models for short-term pv generation forecasting. In: 2016 IEEE International Energy Conference, pp. 1–6 (2016). IEEE

[53] Makridakis, S., Spiliotis, E., Assimakopoulos, V.: Statistical and machine learning forecasting methods: Concerns and ways forward. PloS one **13**(3), 0194889 (2018)

[54] Grogg, K.: Harvesting the wind: the physics of wind turbines. Physics and Astronomy Comps Papers **7** (2005)
